# A Yoga Exercise App Designed for Patients With Axial Spondylarthritis: Development and User Experience Study

**DOI:** 10.2196/34566

**Published:** 2022-06-03

**Authors:** Minh Tam Truong, Obioma Bertrand Nwosu, Maria Elena Gaytan Torres, Maria Paula Segura Vargas, Ann-Kristin Seifer, Marlies Nitschke, Alzhraa A Ibrahim, Johannes Knitza, Martin Krusche, Bjoern M Eskofier, Georg Schett, Harriet Morf

**Affiliations:** 1 Machine Learning and Data Analytics Lab, Faculty of Engineering, Department of Artificial Intelligence in Biomedical Engineering, Friedrich-Alexander University Erlangen-Nürnberg Erlangen Germany; 2 Computer Science Department, Faculty of Computers and Information, Assiut University Assiut Egypt; 3 Department of Internal Medicine 3, Rheumatology and Immunology University Hospital Erlangen Friedrich-Alexander University Erlangen-Nürnberg Erlangen Germany; 4 Rheumatology and Clinical Immunology, Charité Universitätsmedizin Berlin Berlin Germany

**Keywords:** spondylarthritis, digital health application, yoga, usability, patient empowerment, mobile health, mHealth, health applications, smartphone, physical exercise, wellness, mobile phone

## Abstract

**Background:**

Besides anti-inflammatory medication, physical exercise represents a cornerstone of modern treatment for patients with axial spondyloarthritis (AS). Digital health apps (DHAs) such as the yoga app *YogiTherapy* could remotely empower patients to autonomously and correctly perform exercises.

**Objective:**

This study aimed to design and develop a smartphone-based app, *YogiTherapy*, for patients with AS. To gain additional insights into the usability of the graphical user interface (GUI) for further development of the app, this study focused exclusively on evaluating users’ interaction with the GUI.

**Methods:**

The development of the app and the user experience study took place between October 2020 and March 2021. The DHA was designed by engineering students, rheumatologists, and patients with AS. After the initial development process, a pilot version of the app was evaluated by 5 patients and 5 rheumatologists. The participants had to interact with the app’s GUI and complete 5 navigation tasks within the app. Subsequently, the completion rate and experience questionnaire (attractiveness, perspicuity, efficiency, dependability, stimulation, and novelty) were completed by the patients.

**Results:**

The results of the posttest questionnaires showed that most patients were already familiar with digital apps (4/5, 80%). The task completion rates of the usability test were 100% (5/5) for the tasks T1 and T2, which included selecting and starting a yoga lesson and navigating to an information page. Rheumatologists indicated that they were even more experienced with digital devices (2/5, 40% experts; 3/5, 60% intermediates). In this case, they scored task completion rates of 100% (5/5) for all 5 usability tasks T1 to T5. The mean results from the User Experience Questionnaire range from −3 (most negative) to +3 (most positive). According to rheumatologists’ evaluations, attractiveness (mean 2.267, SD 0.401) and stimulation (mean 2.250, SD 0.354) achieved the best mean results compared with dependability (mean 2.000, SD 0.395). Patients rated attractiveness at a mean of 2.167 (SD 0.565) and stimulation at a mean of 1.950 (SD 0.873). The lowest mean score was reported for perspicuity (mean 1.250, SD 1.425).

**Conclusions:**

The newly developed and tested DHA *YogiTherapy* demonstrated moderate usability among rheumatologists and patients with rheumatic diseases. The app can be used by patients with AS as a complementary treatment. The initial evaluation of the GUI identified significant usability problems that need to be addressed before the start of a clinical evaluation. Prospective trials are also needed in the second step to prove the clinical benefits of the app.

## Introduction

### Background

Axial spondylarthritis (AS) is a systemic rheumatic disease that causes chronic inflammation that affects the spinal column and adjacent joints. Affected individuals have inflammatory back pain and decreased spinal mobility because of spinal stiffening. The symptoms are related to pain and decreased function and quality of life. If inflammation progresses, deformities and ossification of the spine may occur. To mitigate the progression of the disease, it is not sufficient to only follow pharmacological and nonpharmacological treatments. Drug options are limited; although with the approval of biologics for the disease, there has been a marked improvement in the treatment therapy. Nevertheless, nonpharmacological therapy, such as physical therapy and self-directed physical exercises, is a complementary part of treatment and can be easily performed by the patients if there is sufficient motivation for physical exercise on the part of the patients [1,2]. Therefore, it is essential to foster active patient participation through self-management interventions, which give patients the ability to actively manage their symptoms and treatment. Active engagement has a positive impact on clinical patient outcome [3]. Digital health apps (DHAs) focusing on exercise can be one way to easily motivate patients in their daily lives [4]. In a survey on the usefulness and willingness to use and pay for a rheumatic self-management app, many patients had a positive attitude toward it. However, the success of the interventions in real life is limited by poor patient-physician coordination and reduced access to self-management programs for patients [5]. These circumstances have particularly worsened since the outbreak of the COVID-19 pandemic.

### Objectives

Health insurance companies in Germany offer various preventive services such as web-based coaching and physical exercise courses [6]. The courses provide a variety of services, ranging from stress management to back and endurance training, and take place at a specific location. Web-based coaching programs are web-based courses and fitness apps that help patients track their diet, improve their physical condition, and provide exercises and information resources on specific topics, such as their back health [7,8]. These services are widely accepted by patients. However, they generally target patients with various musculoskeletal conditions who need support to overcome the challenges of physical inactivity, sedentary behavior, and unhealthy diets. As these offerings tend to be aimed at a broad community, they often overlook the needs of patients with AS or specific information that is important to them. There is a lack of solutions that are aimed exclusively at patients with AS and meet the demands mentioned earlier [9]. The possible solutions are presented below.

Mobile devices, such as smartphones and tablet computers, are widely used by patients with rheumatic diseases and can assist the user in daily living without special training. Mobile health apps installed on mobile devices offer great potential to improve patients’ self-management capabilities [5,10]. No therapeutic DHA for patients with rheumatic diseases could be identified in a recent analysis, although patients clearly stated this need [11].

In a survey about the use [12], preferences, and perceptions of DHAs in the era of COVID-19, the attitudes of patients and rheumatologists changed positively toward the implementation of DHAs (38%) in clinical care. DHA use had increased because of COVID-19 (29%). Most patients (74%) and rheumatologists (76%) believed that DHAs are useful in the management of rheumatic and musculoskeletal diseases and felt confident in using these DHAs (90% and 86%, respectively) [12]. In light of this, it is a problem that currently there are no approved DHAs for rheumatological diseases except for individual apps [11,13].

For instance, one existing app on the market calculates the level of AS in a given patient to measure the severity of the disease [14]. This app additionally provides information on the classification, diagnosis, progression, and treatment of the disease [15]. In addition, there are web solutions that focus on displaying exercises suitable for patients with AS [16] or providing educational information about the disease [17]. Another interesting solution helps people with rheumatoid arthritis to monitor their condition by tracking personal facts (eg, inability to work, morning stiffness, and times of infections) [18]. If we focus on DHAs that mainly cover the fitness sector or are specifically designed for AS, we find 3 competitors. The first is the app Assessment of SpondyloArthritis (ASAS) [19]. In this paper, patients can calculate the Ankylosing Spondylitis Disease Activity Score and Bath Ankylosing Spondylitis Disease Activity Index (BASDAI). In addition, there are informative parts about the disease and questionnaires. The second competitor is the fitness app Gymondo [20]. It focuses on fitness and nutrition. Exercises lasting approximately 50 minutes are designed to prevent back pain. A fitness test can be performed as a bonus. However, there is a lack of special exercises for patients with rheumatic diseases. Another app is Kaia Health, which is specifically aimed at back pain [21] and focuses on pain therapy. Participants receive feedback via pose estimation.

Notwithstanding these, a solution specifically for patients with AS, ideally combining the benefits of fitness exercise, education, and progress tracking, is not yet available.

Nonpharmacological therapy for AS should focus on muscle building and stretching. Yoga combines these 2 aspects and can be adapted for different levels of difficulty. Some studies on patients with rheumatic diseases have shown improvements in function and disease activity. Experience of pain and sleep behavior can also be positively influenced by yoga [22,23]. Moreover, there already exist individual studies investigating the influence of yoga on patients with AS (ClinicalTrials.gov identifier: NCT04281238; DRKS-ID: DRKS00025215 [24]).

This motivated us to develop the app *YogiTherapy* to improve current rheumatology care. The aim of the app is to promote flexible exercising in a home-based environment and to improve the quality of coordination of care between patients and physicians through self-monitoring.

This study is the first step in DHA development and mainly focuses on the technical aspects and the evaluation of the graphical user interface (GUI).

To ensure that the developed app is appropriately tailored to patients’ needs and thus can later be successfully implemented as digital health care technology, it is important to already evaluate its user experience in an early development stage. Schrepp et al [25] define user experience as a holistic concept that combines classical usability criteria with hedonic quality criteria. Therefore, this pilot study aimed to identify usability issues of *YogiTherapy*’s GUI and to evaluate its user experience using the User Experience Questionnaire (UEQ). Clinical feasibility and evaluation were not examined in this study.

## Methods

### Overview

This section highlights the development process and provides an overview of the basic structure of the apps under investigation. Furthermore, it presents the methods to evaluate the user experience as well as digital habits and use of smartphone-based apps.

### Development of the App YogiTherapy

*YogiTherapy* was implemented as a DHA by engineering students from the Machine Learning and Data Analytics Lab at the University of Erlangen-Nuremberg (FAU) and physicians at the University Hospital in Erlangen. Feedback from patients with rheumatic diseases, participating in live yoga classes, was also considered during the conceptional design process. This enabled us to adapt all yoga positions to patients’ movement limitations.

We wanted to create a cross-platform mobile app to target users of the most popular and widely available mobile platforms, iOS and Android [26]. Thus, we chose the open-source cross-platform mobile app framework *React Native*.

We structured the app into 5 sections: training, pose estimation, assessment tests, progress tracker, and information.

**Figure 1:**
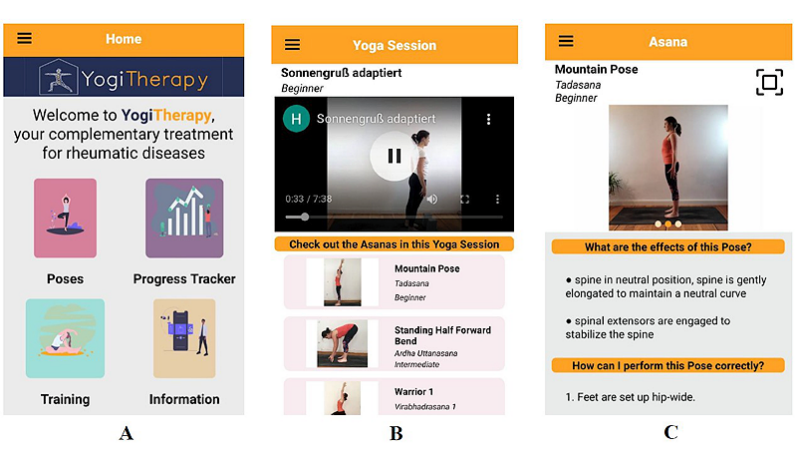
Home screen (A) and training section (B and C) in the app. (B) Video tutorial and its corresponding static yoga poses. (C) Instruction page of a selected yoga pose.

The home screen of the app provides easy access to the poses, training, progress, and information sections (Figure 1A). The following paragraphs present the functionalities of the app.

The purpose of the training section of the app was to provide users with different exercise resources targeted specifically for patients with AS. Compared with other available exercising apps, the yoga poses and instructions were provided by a certified yoga instructor with a professional background in rheumatology to ensure that they are adequately adapted to patients’ needs.

The exercise materials were shared in the form of video tutorials and images. The videos, which can be played directly inside the app (Figure 1B), were divided into beginner and intermediate levels, depending on movement restrictions and fitness level. In addition, the training section offered detailed instructions and visual depictions of each pose (Figure 1C). This allowed the users to learn and perform each pose in advance without sustaining injuries. With the introduction of pose estimation (computer vision techniques that estimate the spatial location of joints from image or video data), we were able to provide users with real-time feedback during the performance of single static yoga poses. The feedback pipeline consisted of 2 steps: pose estimation and pose assessment. The pose estimation relied on a pretrained open-source PoseNet model (version 2.2.1; MobileNet version 2.0.4) that was fed with a live stream of the camera view to detect 17 key points (eg, left ankle) and output spatial information. This spatial information of the detected key points was then compared with the extracted key points of a reference image of the yoga pose using cosine similarity and Euclidean distance. As soon as the cosine similarity between both key point data sets exceeds a defined threshold, the detected skeleton in the camera view turns green to indicate good accordance with the reference pose image. In the case of low similarity, a red-colored skeleton signals the user to keep adjusting the pose.

**Figure 2:**
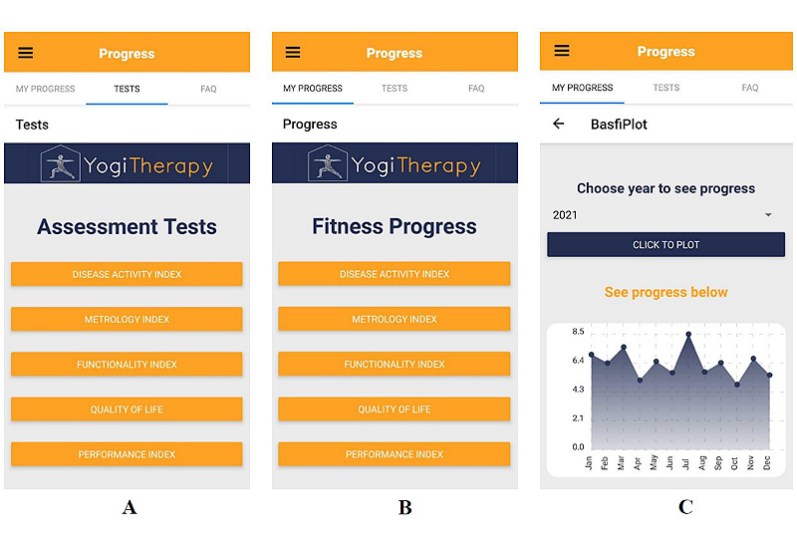
Progress tracker section in the app. (A) Overview screen of assessment tests. (B) Overview screen for test results. (C) Visual depiction of the annual test result of the Bath Ankylosing Spondylitis Functional Index test.

We incorporated a progress tracker to give users the possibility to self-monitor their symptoms and disease status and track their own progress over time (Figure 2). This allows users to take different assessment tests (a detailed explanation of the chosen assessment tests is provided in Multimedia Appendix 1 [27-29]) and then review their health progress over time.

We included 3 subjective test instruments, the Bath Ankylosing Spondylitis Functional Index (BASFI), the BASDAI, and the Ankylosing Spondylitis quality of life, and 2 objective test instruments, the Bath Ankylosing Spondylitis Metrology Index (BASMI) and the Ankylosing Spondylitis Performance Index.

Exactly how often these tests need to be administered to collect sufficient reliable data over a long treatment period will be the subject of a prospective clinical evaluation. The test results were stored locally on a mobile device to comply with high data privacy standards. This personal medical record can be shared with consulting rheumatologists at follow-up appointments if desired. Patients can access and view their entire medical history on a progress tracker dashboard. The dashboard was structured according to the type of test (Figure 2B), so that it was possible to display the monthly means for each test in the form of an annual line or bar chart (Figure 2C).

For this study, we only worked with the Android version of the system and delivered it in the package file format Android Package (APK).

### Ethics Approval

All participants provided consent, and the study was approved by the ethics committee of the Medical Faculty of the University of Erlangen-Nuremberg, Germany (Antrag 8_ 21 B).

### Study Design

For this pilot user experience evaluation study, we used a mixed methods design and collected qualitative and quantitative data. Participants performed a formative usability test immediately followed by a posttest questionnaire to quantify the user experience of *YogiTherapy*’s GUI. This pilot study was conducted in March 2021, and 10 subjects were recruited within 2 weeks through the Department of Rheumatology and Immunology at the University Hospital Erlangen. Participation was voluntary, and there was no preselection in the form of screening. To be included in the study, participants had to be diagnosed with a rheumatic disease or be a rheumatologist. We chose to not limit patient recruitment to only patients with AS because this study did not aim to investigate the clinical feasibility or benefits of the app as a DHA for patients with AS but to evaluate the GUI during the continuing development process. Moreover, we aimed to evaluate the app design from all target users’ perspectives. This includes not only patients but also rheumatologists, who have a special mediating role and can take advantage of the continuous health data provided by the app to adjust the treatment. Therefore, we deliberately recruited 5 patients and 5 rheumatologists to explore the differences in the measured user experience between the 2 main target user groups. The general exclusion criterion was lack of basic English skills, as it is the main language of the first app prototype.

This pilot study was part of the first development cycle and served as a first insight into the app’s user experience. Previous research estimates that approximately 7 to 8 participants in a usability study can uncover approximately 80% of the existing usability issues when each individual participant uncovers an average of 20% of the issues. Usability studies in later development iterations require a larger sample size because it is becoming increasingly difficult to detect issues [30].

Owing to the COVID-19 restrictions and time limitations, one half of the study was conducted in person and the other half of the study was conducted remotely. The patients’ group participated in this study, which was conducted at the outpatient department of the Rheumatology and Immunology of the University Hospital Erlangen, whereas the rheumatologists’ group exclusively followed the remote study procedure.

In the beginning, each subject was informed about the purpose and process of the study and was asked to provide written consent. The participants were provided with a test smartphone (Google Pixel 3a, Google Inc) on which the app *YogiTherapy* was preinstalled. In the case of the remote study, the procedure participants could either borrow the test smartphone or install the app on their private Android device.

The study procedure was divided into 2 phases. In phase 1, patients with a rheumatological disease and a rheumatologist were given some time (approximately 3 minutes) to explore the app of *YogiTherapy* and its specifications. After exploration, the patients were instructed to complete 5 navigation task scenarios in the app, which were derived from a prospective user context. The task scenarios were as follows:

- T1. Select a yoga video and start the video.
- T2. Go to the information page about nutrition.
- T3. You have been given a Metrology Index (BASMI) of 7.5 by your doctor. Enter the result in the app and save the result.
- T4. Visualize the Metrology Index (BASMI) over time in the form of a graph.
- T5. Take a test to find out your Disease Activity Index (BASDAI) and save your result.

Patients and rheumatologists could skip each task if they experienced difficulties. For each task, the binary outcome (success or fail) was self-reported using a web-based posttest survey. The task completion rate (TCR), which is the percentage of participants who completed the task, was derived from this [22].

Throughout the interaction with the app, the participants were asked to follow the think-aloud protocol and communicate their thoughts, actions, expectations, and observations. The study conductor collected qualitative data by taking field notes on the subject’s verbal reflections. The think-aloud protocol for rheumatologists could not be produced within the framework of the remote study.

In posttest phase 2, each test person was administered a web-based UEQ to assess the user experience of the tested product. In addition, the questionnaire contained questions regarding task completion, aspects of the app they liked or disliked, demographics (sex, age, and patient or rheumatologist), and smartphone experience and habits.

The UEQ allows a quick and reliable measurement of the user experience of interactive products. The questionnaire consists of 6 scales with 26 items in total that measure the distinct quality criteria of user experience. It provides answers to the following questions:

- Attractiveness: Do users like or dislike *YogiTherapy*?
- Perspicuity: Is it easy to get familiar with *YogiTherapy*? Is it easy to understand how to use *YogiTherapy*?
- Efficiency: Can users solve their tasks quickly and efficiently? Does the user interface of *YogiTherapy* look organized?
- Dependability: Does the user feel in control of the interaction? Is the interaction with *YogiTherapy* secure and predictable?
- Stimulation: Is it exciting and motivating to use *YogiTherapy*? Does the user feel motivated for the further use of *YogiTherapy*?
- Novelty: Is the design of the product innovative and creative? Does *YogiTherapy* catch the user’s attention?

The attractiveness scale captures the user’s general impression of the app and is influenced by the remaining 5 scales. The scales perspicuity, efficiency, and dependability provide information about pragmatic (goal-directed) quality aspects, whereas stimulation and novelty describe hedonic (nongoal-directed) quality criteria [25]. The items have the form of a semantic differential, that is, each item is a pair or opposites with a 7-point Likert scale [31,32].

### Analysis

The quantitative participant feedback obtained from the UEQ was processed and analyzed using the Excel Data Analysis Tool [33] provided by the authors of the questionnaire. Independent samples 2-tailed *t* tests (assuming unequal variance, α=.05) were used to compare the user group differences for mean values of each scale. To determine the relative strengths and weaknesses of *YogiTherapy*, the combined UEQ results of both participant and user groups were compared with a benchmark data set containing 246 user experience evaluations of established products [31]. The TCR of the usability test, demographic characteristics, and smartphone use characteristics were examined using a descriptive analysis. Qualitative data obtained from the think-aloud protocol were clustered in terms of the usability problems encountered.

## Results

### Descriptive Information

In total, 10 participants completed the study procedure and were included in the final analysis. The participant group *Patient* consisted of 5 patients (3 females and 2 males) with a mean age of 59.4 (SD 6.2) years. Furthermore, the patients were diagnosed with axial spondylarthritis, psoriatic arthritis, and rheumatoid arthritis. In the participant group *Rheumatologist*, 5 rheumatologists were recruited with a mean age of 38.8 (SD 10.7) years.

### Results of Group Analysis

The TCR values obtained from the formative usability tests for both groups are presented in Table 1. All rheumatologists stated that they had successfully completed all tasks, whereas the patient group particularly struggled with tasks related to the *Progress Tracker* screen in the app. Of the 5 patients, 2 (40%) were unable to complete tasks T3 and T4. Table 2 presents the issues identified using the think-aloud protocol during the patients’ interaction with the app. The identified problems were in accordance with the low completion rates for tasks T3 to T5.

**Table 1:** Task completion rates of both participant groups.

Tasks	Task completion rate, n (%)	
	Patients (n=5)	Rheumatologists (n=5)
T1	5 (100)	5 (100)
T2	5 (100)	5 (100)
T3	3 (60)	5 (100)
T4	3 (60)	5 (100)
T5	4 (80)	5 (100)

**Table 2:** Usability issues identified by patients’ think-aloud protocols.

Aspect	Usability issue
Overall	German version would be easier to handle App crashes sometimes Information pages are too text-heavy Filter list function should support automatic filtering Asana list under video was mistaken for an automatic playlist because of design A smartphone is impractical for exercising, and a tablet would be more appropriate, especially for the videos
Progress tracker	Top tab navigation (My Progress—Tests-FAQ^a^) was overlooked Difficulties finding the tab Tests because the default landing page of the Progress Tracker is My Progress and both the tabs look identical (compare Figure 2A and Figure 2B)

^a^*My Progress-Test-FAQ* is the tab where patients can see the disease progress as it was requested in T4. The Metrology Index (BASMI) is visualized over time in the form of a graph.

**Figure 3:**
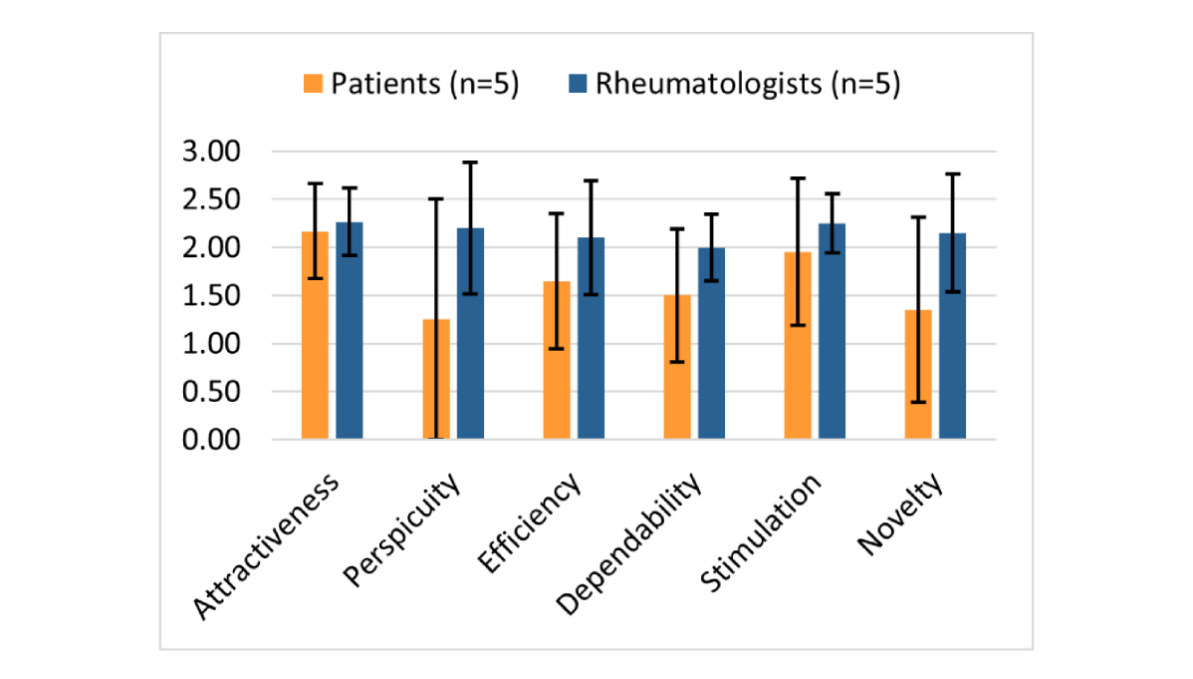
User Experience Questionnaire scale means depending on the participant group. The error bars represent the 95% CIs of the scale mean.

In the posttest phase, we asked about their experiences and habits using digital apps and smartphones. Overall, 80% (4/5) of the patients considered themselves to be experienced in using these technologies, compared with 20% (1/5) of the beginners. Majority (4/5, 80%) of the patients reported a regular habit of using smartphones and tablets for 4 to 7 days per week. In the rheumatologist group, 100% (5/5) rheumatologists used their smartphone and tablets for 4 to 7 days per week. Moreover, 40% (2/5) of them claimed to be experts in using these technologies, and 60% (3/5) of them self-reported intermediate skills. The participants’ feedback was measured using the UEQ, as shown in Figure 3.

Table 3 provides the UEQ results at the 95% CIs. The scale ranges from −3 (most negative evaluation) to +3 (most positive evaluation) [25]. The mean score of both groups was >0.8, indicating a positive evaluation. Rheumatologists rated *YogiTherapy*’s attractiveness, pragmatic, and hedonic qualities equally high, whereas patients found the app very attractive but reported much lower pragmatic and hedonic qualities. The lowest mean score was for the scale perspicuity (mean 1.250, SD 1.425) in the patient group. The most striking observation to emerge from the data comparison was that, on average, the rheumatologists group rated each user experience scale higher than the patient group. The mean score of rheumatologists was 2.0, indicating a very positive evaluation. However, the differences in the means for each UEQ scale between the groups were not statistically significant.

**Table 3:** User Experience Questionnaire scales of both participant groups: patients and rheumatologists.

Scale	CIs (*P*=.05) per scale					
	Patients (n=5)			Rheumatologists (n=5)		
	Value, mean (SD)	Confidence	95% CI	Value, mean (SD)	Confidence	95% CI
Attractiveness	2.167 (0.565)	0.495	1.671-2.662	2.267 (0.401)	0.352	1.915-2.618
Perspicuity	1.250 (1.425)	1.249	0.001-2.499	2.200 (0.779)	0.682	1.518-2.882
Efficiency	1.650 (0.802)	0.703	0.947-2.353	2.100 (0.675)	0.592	1.508-2.692
Dependability	1.500 (0.791)	0.693	0.807-2.193	2.000 (0.395)	0.346	1.654-2.346
Stimulation	1.950 (0.873)	0.765	1.185-2.715	2.250 (0.354)	0.310	1.940-2.560
Novelty	1.350 (1.098)	0.963	0.387-2.313	2.150 (0.698)	0.612	1.538-2.762

**Figure 4:**
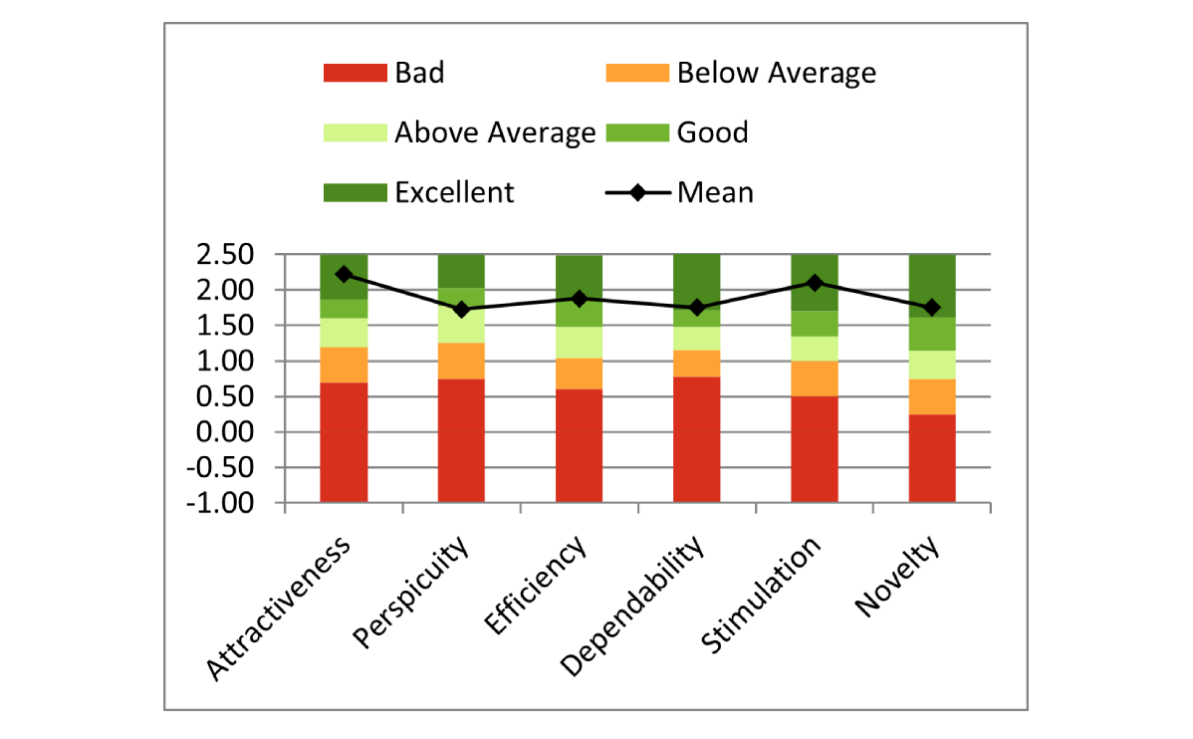
*YogiTherapy*’s mean User Experience Questionnaire scores (n=10) compared with the benchmark data set.

The results of benchmarking are shown in Figure 4. In comparison with established interactive products, *YogiTherapy*’s overall attractiveness, stimulation, novelty, and dependability were classified as excellent. In terms of efficiency, the app achieved the category good. The worst results were achieved for the scale perspicuity (mean 1.725, SD 1.193). Here, *YogiTherapy* was above average compared with the benchmark data set.

## Discussion

### Principal Findings

Overall, the findings of this pilot study were encouraging. *YogiTherapy*’s general attractiveness and hedonic qualities scored high compared with the products in the benchmark data set, suggesting that the app fulfilled users’ general expectations in terms of attractiveness, stimulation, and novelty. Furthermore, the benchmark identified pragmatic qualities as the weaknesses of GUI. Compared with established interactive products, it only achieved the category *Good* and *Above Average* for the scales efficiency and perspicuity. As general user experience has grown over time, new products should reach the category *Good* on all scales [31]. The poor efficiency and perspicuity scores can be attributed to patient evaluation.

Their lower ratings for pragmatic qualities may be related to the assigned usability tasks. The associated TCR indicated that the patient group struggled with all tasks related to the *Progress Tracker* section in the app (Table 1). This is also evident from their think-aloud protocols; the poorer success on the tasks might be explained by inadequate English skills and a confusing user interface in the *Progress Tracker* section. Participants under observation felt very insecure about their English and stated that using the app would be much more pleasant and easier if it were in their native language, German. Furthermore, the 3-part tab navigation structure of the *Progress Tracker* screen and the identical design of the tabs *My Progress* and *Tests* led to confusion. All these points might have influenced the participants’ views of the app’s perspicuity and efficiency. The better performance of rheumatologists on the usability tasks may be attributed to their lower mean age and familiarity with digital apps, as they reported using mobile devices on a daily basis. This might also have a positive impact on their English proficiency, which, in turn, would enable self-confident navigation in the app. As perspicuity and efficiency, alongside attractiveness and stimulation, play a key role in successful adherence to DHAs, the goal should be to reach the *Excellent* category on all these user experience scales. The benchmark and qualitative data clarified that pragmatic qualities must be improved to achieve this objective. We see the results as concrete starting points for optimizing the app in further development iterations. This could be achieved by offering a multilingual app that supports the user’s native language and a more illustrated GUI. Icons easily capture the user’s attention, are generally comprehensible, and provide essential information on the first sight. In addition, the user interface elements used for navigation in the *Progress Tracker* section require significant revision, so that the tab navigation is more predominant. The revised app should then be evaluated again in a usability test with a larger sample size and additional task scenarios.

### Comparison With Prior Work

As stated in the Introduction section, currently there are 3 competitors in the category of back pain and digital apps for physical activity. In ASAS app, patients can calculate their disease activity; however, exercises that can be performed at home were missing in this app. To date, no clinical trial has evaluated the ASAS app along with physical exercise. Gymondo provides the option to perform exercises for back pain; however, there is a lack of focus on rheumatologic conditions, specifically AS. In addition, Gymondo is a fitness app that has no option for controlling or managing diseases. Therefore, no clinical studies have been conducted using this app. Similar to Gymondo, there is no way to collect and track disease progression through questionnaires in the Kaia Health app. The exercises are also designed for back pain, but they have no relation to rheumatological diseases. As this app focuses primarily on pain, it has already been evaluated in a number of studies. Similar to *YogiTherapy*, patients were asked for feedback, which is very important for the further development of the app [34]. A clinical trial of 180 participants with nonspecific lower back pain demonstrated that the use of the app over 12 weeks resulted in a significant reduction in pain [35].

### Strength and Limitations

This pilot study had several limitations. First, it only included a small sample size, which affected the CIs of the UEQ scale means. Therefore, all results from the UEQ need to be interpreted with caution. Second, because of limitations resulting from the COVID-19 pandemic (time, staff, and restrictions), it was not possible to create the same experimental environment and conditions for both participant groups. Rheumatologists followed the remote procedure and self-managed the usability test on either their private smartphone or the borrowed test device. No direct observation was possible, and it remains unclear whether the participants conducted the navigation tasks seriously. Therefore, there was no think-aloud data for the rheumatologist group. Third, in this study, the conductor took field notes instead of audio recordings for the think-aloud protocol. Therefore, it was not possible to perform a comprehensive analysis of the transcribed data. Fourth, this pilot user experience study with a clear focus on the technical aspects of the app included patients with various rheumatic diseases

as participants instead of exclusively recruiting patients with AS for the patient group. For use in clinical trials, the app must be directed to patients with AS. Finally, the patients were not native English speakers, which could have affected their ratings. The German version is currently being developed.

### Conclusions and Future Directions

The newly developed and evaluated DHA *YogiTherapy* can be used as a complementary treatment for patients with rheumatological diseases, particularly AS. In contrast to digital apps targeting back pain or physical activity, the designed app is specifically tailored to the needs of rheumatic patients, as it combines physical yoga exercises to improve mobility and continuous self-monitoring of disease progression. The results of the user experience evaluation suggested that *YogiTherapy*’s GUI convinced patients as well as rheumatologists with its innovation, attractiveness, novelty, and dependability. However, it also revealed major usability issues related to the efficiency and perspicuity of the user interface. These will need special attention during revision and reevaluation. Prospective longitudinal clinical trials with a large cohort of patients with AS are needed to assess the actual benefits, safety, and acceptance of the app as well as to evaluate the remote delivery mode of physical exercises compared with face-to-face courses.

## Appendix

Multimedia Appendix 1 Assessment tests used in the *YogiTherapy* app to detect progress in mobility, disease activity, and function through Yoga exercises.

## Abbreviations

AS: axial spondyloarthritis
ASAS: Assessment of SpondyloArthritis
BASDAI: Bath Ankylosing Spondylitis Disease Activity Index
BASFI: Bath Ankylosing Spondylitis Functional Index
BASMI: Bath Ankylosing Spondylitis Metrology Index
DHA: digital health app
GUI: graphical user interface
TCR: task completion rate
UEQ: User Experience Questionnaire

## Data Availability

Raw data will be uploaded and freely accessible.
