# Supplementary material for: A Yoga Exercise App Designed for Patients With Axial Spondylarthritis: Development and User Experience Study
Source: JMIR Form Res. 2022 Jun 3;6(6):e34566. doi: 10.2196/34566 (PMC9206208; doi:10.2196/34566)
Supplement: Multimedia Appendix 1 [file formative_v6i6e34566_app1.docx]

**Supplementary Material**

The Assessment Tests

The patient-reported and disease-specific Bath AS Functional Index (BASFI) is used to evaluate physical functioning in patients with AS as recommended by the Assessment of SpondyloArthritis Society (ASAS) [15]. BASFI includes ten questions regarding activities of daily living such as getting up from a chair or bending and the ability to cope with everyday life. Each question is answered on an 11-point numeric rating scale (NRS), anchored by easy (0) and impossible (10). The item scores are summarized and presented as the mean BASFI score (0–10, 10 = most limited physical function). BASFI is a reliable, valid, and responsive measure of physical function in patients with AS [27-28].

To meet the shortcomings of BASFI, a performance-based test was derived from BASFI: the AS Performance-Based Improvement (ASPI). ASPI is reported to be easy to administer, well tolerated by patients with varying limitations in physical functioning, and feasible in daily clinical practice. It consists of three tests:

1) bending and picking up pens from the floor,

2) putting on socks, and

3) getting up from the floor.

Patients are instructed to perform all tests as quickly as possible, though in a safe manner. The outcome of the performance test is the time needed to complete the tasks, measured in seconds. Afterward patients rate their pain and exertion felt during the performance test on a numerical scale. ASPI is a reliable, valid, and responsive method to evaluate changes in the physical function of patients with AS [15].

Another important topic to be taken into consideration is the assessment of mobility. This can be successfully obtained from Bath AS Metrology Index (BASMI), which includes four measures of spinal mobility (lateral spinal flexion, cervical rotation, lumbar flexion, tragus-to-wall distance) and one measure of hip mobility (intermalleolar distance). BASMI was developed to quantify the mobility of the axial skeleton in patients with AS and identify clinically significant changes in the spinal movement [29]. The BASMI score is usually measured by a clinician since it requires a high level of precision and adaptations to the patient's individual posture and circumstances [29]. For this reason, we decided to not use computer vision to automatically measure the BASMI score and stuck with the conventional approach instead. Thus, patients can enter and save their score estimated by the physician.

In addition, we included a very common evaluation tool for AS, the Bath Ankylosing Spondylitis Disease Activity Index (BASDAI). BASDAI is a self-administered instrument with six questions, involving individual domains regarding the following topics: fatigue, spinal pain, joint pain, swelling, areas of local tenderness, and morning stiffness. Each item has a Visual Analogue Scale (VAS) ranging from 0 (none) to 10 (very severe). Item six (morning stiffness, duration) is measured by a time scale (0–2 h). The summed score of items five (morning stiffness, severity) and six is converted to a 0–10 scale, with a lower score indicating less disease activity [27]. The Ankylosing Spondylitis Quality of Life Questionnaire (ASQoL) was specifically developed to assess the quality of life in AS patients. This instrument is highly appreciated by patients, sensitive to change over time, and feasible. The questionnaire contains 18 questions about quality of life. Each statement on the ASQoL is given a score of “1” or “0”. A score of “1” is given where the item is affirmed, indicating adverse QoL. All item scores are summed to give a total score or index. Scores can range from 0 (good QoL) to 18 (poor QoL) [15].
